# Evidence of platelet activation in multiple sclerosis

**DOI:** 10.1186/1742-2094-5-27

**Published:** 2008-06-27

**Authors:** William A Sheremata, Wenche Jy, Lawrence L Horstman, Yeon S Ahn, J Steven Alexander, Alireza Minagar

**Affiliations:** 1 Multiple Sclerosis Center and Department of Neurology Miller School of Medicine, University of Miami, Miami, Florida, USA; 2 Wallace Coulter Platelet Laboratory, Division of Hematology and Oncology, Department of Medicine, Miller School of Medicine, University of Miami, Miami, Florida, USA; 3 Department of Cellular and Molecular Physiology, Louisiana State University Health Science Campus, Shreveport, Louisiana, USA; 4 Department of Neurology, Louisiana State University Health Science Campus, Shreveport, Louisiana, USA

## Abstract

**Objective:**

A fatality in one multiple sclerosis (MS) patient due to acute idiopathic thrombocytopenic purpura (ITP) and a near fatality in another stimulated our interest in platelet function abnormalities in MS. Previously, we presented evidence of platelet activation in a small cohort of treatment-naive MS patients.

**Methods:**

In this report, 92 normal controls and 33 stable, untreated MS patients were studied. Platelet counts, measures of platelet activation [plasma platelet microparticles (PMP), P-selectin expression (CD62p), circulating platelet microaggragtes (PAg)], as well as platelet-associated IgG/IgM, were carried out. In addition, plasma protein S activity was measured.

**Results:**

Compared to controls, PMP were significantly elevated in MS (p < 0.001) and CD62p expression was also markedly elevated (p < 0.001). Both are markers of platelet activation. Platelet-associated IgM, but not IgG, was marginally elevated in MS (p = 0.01). Protein S in MS patients did not differ significantly from normal values.

**Conclusion:**

Platelets are significantly activated in MS patients. The mechanisms underlying this activation and its significance to MS are unknown. Additional study of platelet activation and function in MS patients is warranted.

## Background

The fatal outcome in one of two multiple sclerosis (MS) patients with idiopathic thrombocytopenic purpura (ITP) prompted our interest in platelet activity and function in the context of MS. Although Putnam investigated a possible role of venule thrombosis as a factor in central nervous system (CNS) demyelination in 1935 [1], a role for platelets in CNS demyelination was not further considered until a series of papers in the 1960s, such as that of Wright et al. [2] For example, Nathanson and Savitsky [3] employed a measure of platelet adhesiveness in 132 subjects, 60 of whom had MS. The investigators reported increased platelet adhesiveness in both MS and Guillain-Barre correlating with disease activity. Although other investigations confirmed their findings, they contributed little additional information. More recently, a central role for platelets in inflammation has emerged, as reviewed [4,5].

Our observation of platelet abnormalities in MS [6] and subsequent observation of thrombosis in cutaneous venules and capillaries adjacent to subcutaneous ulcers complicating subcutaneous injections of interferon-beta1b [7] heightened our interest in a possible role of platelet dysfunction in MS. To investigate the basis of these observations, we have applied the flow cytometric analysis of platelet-derived microparticles (PMP) and CD62p expression, as well as other more conventional assays. For this study, we employed consecutively recruited patients and measured, in addition to routine tests such as platelet counts, the expression of platelet activation marker P-selectin (CD62p), platelet microparticles (PMP) in plasma, platelet micro-aggregates (PAg), protein S activity, and platelet-associated immunoglobulins IgG and IgM, as described following.

## Methods

### Patient population

Thirty-three treatment-naïve, clinically stable relapsing-remitting MS patients and 92 normal control subjects were recruited. The study protocol was approved by the IRB office of University of Miami and signed informed consents were obtained.

### Blood sampling

A 4.5 mL blood sample was drawn into Vacutainer^® ^tubes containing citrate, using a 21-gauge butterfly needle following light application of a tourniquet. After blood flow was established, the tourniquet was promptly removed to minimize artifactual platelet activation. The first tube drawn was not used for platelet studies to avoid platelet activation from thromboplastin released by the puncture wound. Samples were prepared for flow cytometry not more than 2 hours after phlebotomy. Although drawing into Vacutainers induces slight platelet activation compared to the syringe method, they were required by the phlebotomy clinic, and normal controls were drawn in the same way.

### Platelet counts and protein S assay

Platelet counts and plasma protein S activities were carried out by the clinical pathology laboratories, University of Miami. Normal ranges of values were used for reference.

### Platelet microparticle (PMP) assay

The method as described by Jy et al. [8,9] was employed with minor modifications [10,11]. Briefly, platelet-rich plasma (PRP) was prepared by centrifuging whole blood 10 min. at 160 × g. Platelet-poor plasma (PPP) was then prepared by centrifuging PRP for 6 min. at 2000 × g. Five μL of fluorescein isothiocyanate (FITC)-conjugated anti-CD41 was added to 20 μL of PPP, and after 20 min., 25 μL of 4% PFA fixative also added. After 20 min. fixation, 2.0 mL of PBS was added and PMP were measured by flow cytometry with the neutral density filter removed. Events were detected and counted by triggering on the fluorescent signal. Results are expressed as PMP × 10^7^/μL plasma. Particle counts measured in 1-minute runs were converted to concentrations in plasma based on the dilution factor and rate of flow through the cytometer, as detailed [12].

### Platelet aggregates (PAg)

Circulating PAg were measured by flow cytometry, as described [13,14]. This assay reflects the tendency of activated platelets to form micro-aggregates in circulation.

### Platelet activation marker P-selectin (CD62p)

Platelet activation marker P-selectin (CD62p) was measured by flow cytometry using phycoerythrin (PE)-labeled anti-CD62p together with platelet marker, anti-CD41, as described [15,16]. Briefly, to 5 μL of citrated whole blood was added 100 μL PBS, and then 5 μL of each mAb. After 10 min, 160 μL of 4% PFA in PBS was added. Following 10 min. fixation, 1.0 μL of PBS was added, and after 2 hours hemolysis the sample was ready for cytometry. All samples were counted for 1 minute.

### Platelet-associated IgG, IgM

Platelet associated immunoglobulin was measured as described [17], except that the F(ab)_2 _fragment of goat anti-human IgG and IgM was employed to reduce non-specific Fc-dependent binding, thus increasing accuracy of the measurement. In our method, fresh platelets are isolated in the presence of 2 mM EGTA and 2 μM prostaglandin E_1_, preventing activation and emergence of internal IgG and IgM on the platelet surface, thereby avoiding objections sometimes raised regarding measurement of platelet-associated IgG and IgM.

## Results

### Platelet counts

**Figure 1:**
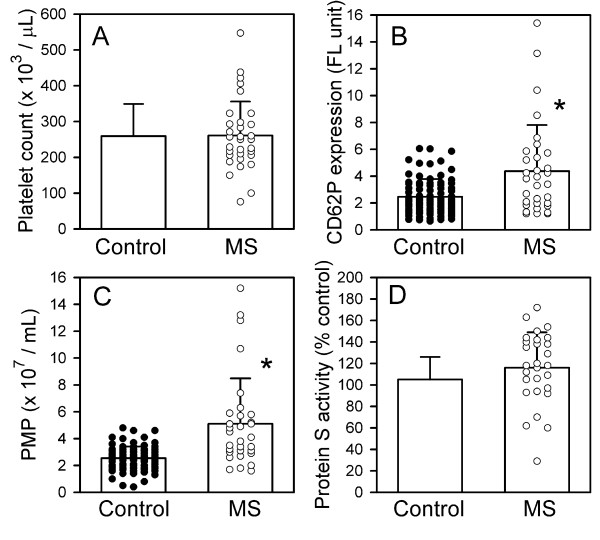
**Comparison of platelet count, platelet activation marker CD62P expression, PMP, and protein S between controls and MS patients**. **(A) **Mean platelet counts did not differ between the MS and control groups. **(B) **Elevation of platelet activation marker CD62p in the MS group was highly significant, *P < 0.001. Not shown or included in the analysis was one extremely high value (60.6), possibly a technical error. **(C) **PMP counts in the MS patients were significantly higher than controls, *p < 0.001. Not shown or included in the analysis was one very high outlying value (25.2). **(D) **Protein S activity (% control) did not differ significantly between MS and controls. All values are expressed as mean ± SD. The institutional control values (mean ± SD) of platelet count and protein S activity were obtained from the laboratory of University of Miami Hospital and Clinics, and are not shown as individual dots because the numbers are too large.

Mean platelet counts for the n = 33 patients were within the normal range (261 ± 95 × 10^3^/μL, ± S.D.), as compared with the reference value for the normal controls (259 ± 90 × 10^3^/μL) studied concomitantly; p = NS (Figure 1A). However, 7 MS patients had platelets counts/μL <200,000 and 3 had counts ≤150,000 (76,000, 100,000, 150,000), consistent with our experience that mild thrombocytopenia is not uncommon in MS patients.

### Platelet activation marker CD62p

The mean fluorescence intensity of CD62p, a marker of platelet activation, was also elevated in MS as compared to controls: mean values ± SD were 4.4 ± 3.6 for the MS and 2.5 ± 1.3 for the controls; p < 0.001 (Figure 1B).

### Platelet microparticles (PMP)

In the MS group, PMP were elevated by approximately a two-fold margin compared with the controls (Figure 1C). The mean value for the MS patients was 5.1 ± 3.4 (× 10^7^/mL, ± SD), compared to 2.5 ± 0.9 for controls; p < 0.001. These PMP are released into the plasma in the process of platelet activation and are known to exhibit procoagulant activity. This is accomplished by supplying a suitable phospholipid surface for assembly of the factor X-ase and factor II-ase (prothrombinase) complexes, known as platelet factor 3 (PF3) activity [18], and by carrying tissue factor (TF) [19,20].

### Platelet micro-aggregates (PAg)

Circulating platelet micro-aggregates, an additional marker of platelet activation, was also elevated in the MS group, relative to controls, p = 0.01, to a degree similar to the CD62p (data not shown).

### Protein S activity

No significant difference was found between the MS and control groups in this measure (Figure 1D). Values were, ± SD, 105 ± 21% for controls and 116 ± 33% for MS patients.

### Platelet surface-associated IgG, IgM

**Figure 2:**
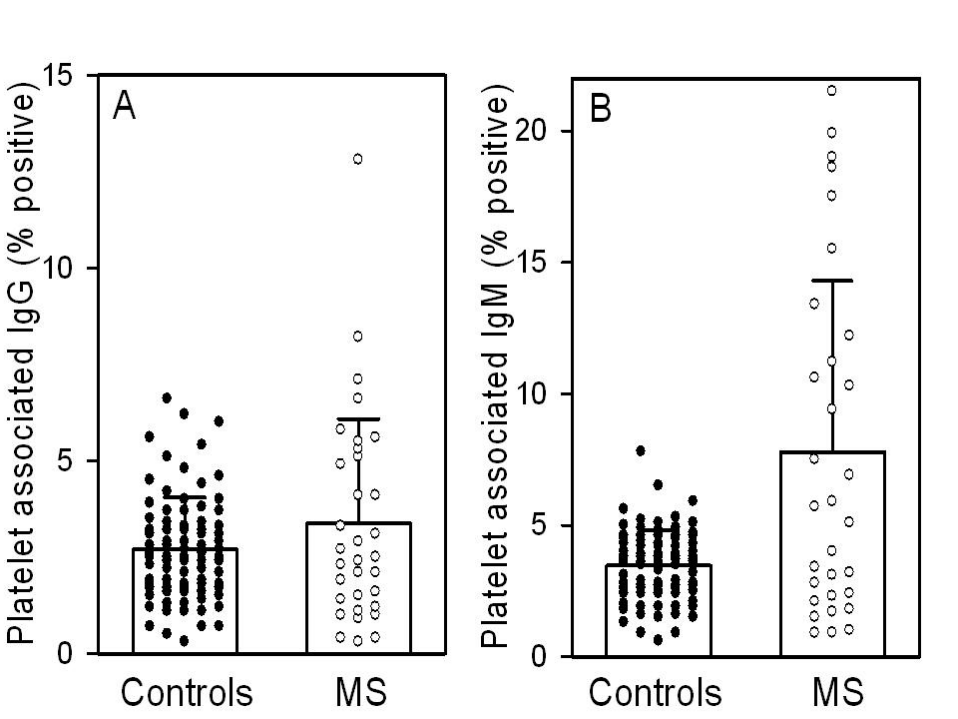
**Comparison of platelet-associated IgG and IgM between controls and MS patients**. Results are expressed as percentage of platelets positive for the indicated immunoglobulin, where positive is defined as >3SD above the mean for normal subjects in this laboratory by the procedure referenced in the methods section. **(A) **Results for platelet-associated IgG showed no significant difference between the MS group and controls. Value were, ± SD: 2.7 ± 1.3 for controls vs. 3.4 ± 2.7. **(B) **In contrast, mean platelet associated IgM was significantly higher in the MS group, *p < 0.01. Values were, ± SD: 3.4 ± 1.3 for controls vs. 7.8 ± 6.5 for MS. Not shown or included in analysis were 2 very high outlying values (55.9, 65.9).

Immunoglobulin IgM associated with platelets was significantly elevated in the MS group (7.8 ± 6.5 percent cells positive) as compared to controls (3.4 ± 1.3), p < 0.01 (Figure 2B). However, IgG was not increased (3.4 ± 2.7 in MS vs. 2.7 ± 1.3 in controls) (Figure 2A). This IgM may represent immune complex formation, autoantibody, or it could reflect chronic platelet activation. Chronic platelet activation has been reported to cause externalization of immunoglobulins normally found within platelets. Surface expression of IgM may sensitize the platelet for destruction by complement [10], or alternatively, by the reticuloendothelial system.

## Discussion

By all three measures (PMP, PAg, CD62p), we have documented the presence of platelet activation in MS patients. Increased PMP and increased CD62p expression are accepted markers of platelet activation [15,21]. Our observations strongly support the conclusion that platelets are chronically activated in MS, and support results in the earlier literature [2].

Platelet activation may be an epiphenomenon consequent to the disease process in MS, possibly secondary to endothelial injury. Indeed, we have reported elevated plasma endothelial microparticles (EMP) in MS, positive for platelet endothelial cell adhesion molecule-1 (CD31/PECAM-1) [22] and suggested that this increase in EMP may reflect the interaction of activated T-cells with endothelium. It is also possible that interaction of released PMP with underlying endothelium contribute**s **to the endothelial abnormalities involved in the pathophysiology of MS. Binding of P-selectin on PMPs to P-selectin glycoprotein ligand-1 (PSGL-1) and PECAM-1 on lymphocytes is known to induce or increase the expression of integrins such as integrin α4β1 (VLA-4), and thus facilitate lymphocyte binding to endothelium [23]. However; the *in vivo *role of CD62p+ PMP and CD31+ EMP and their interactions in MS remains unknown. It is known that PMP can transport a variety of bioactive agents including platelet activating factor (PAF), amyloid precursor protein, complement factors, and other molecules [24,25], all of which could contribute to the disease process in some manner.

Although study of the role of cell-derived microparticles in disease processes is still in its infancy, it is clear that PMP are involved in angiogenesis [26,27] and in certain malignant processes [28,29]. In light of these developments, it is reasonable to postulate that PMP may play an active role in the pathogenesis of MS. Several chemokines have been detected on platelets [30] and some are carried by PMP [31,32]. The chemokine receptor CXCR4 can be transferred by PMP from cell to cell [33]. Leukocyte activation by PMP has been also reported [34], illustrating another potential link between PMP and the inflammatory disease process.

It remains unclear how platelet activation and the release of PMP pertain to our clinical observation of 2 cases of ITP associated with MS at our center. This finding, in accord with the earlier literature [2], suggests the presence of a procoagulant state in MS, and elements of the coagulation system such as fibrin and tissue factor (TF) are found in MS lesions [35]. However, review of the available literature has identified additional MS patients with ITP complicating their illness. Granier et al in 2001 reported a 40 year old French patient with well-documented MS who had a transient thrombocytopenia [36]. Four additional cases have been identified recently in Spain [37]. However, two earlier cases [1997] with Hodgkin's and thrombocytopenia cannot be accepted as ITP [38]. Recent epidemiological studies in Maryland [39] and in Nova Scotia, Canada [40] have brought to light additional cases. Segal et al. identified 4 such cases in children [39] and Kirby et al. have identified 22 MS patients with "immune thrombocytopenia" [40]. Segal's report reflects a 25-fold higher incidence of ITP in MS in the Maryland study and, although details from the Canadian study are not available, an even higher coincidence may occur there. Although PMPs were thought to possess solely procoagulant properties, it appears that some PMP phenotypes may exert predominantly anticoagulant functions by modulating the protein C/S system [41].

Platelet associated IgM was increased in the MS group (p < 0.01) but platelet associated IgG was not. Our finding of elevated platelet-associated IgM and platelet activation may reflect the presence low levels of serum anti-phospholipid antibodies in MS. This finding is consistent with reports of predominately IgM anti-phospholipid antibodies in MS. Sugiyama et al found 14 of 32 patients positive for IgM anti-phospholipid antibody but only 3 of 32 positive for IgG [42]. Using another method, we have found anti-phospholipid antibodies exclusively of IgM class in MS [43]. Patients with the anti-phospholipid syndrome are prone to recurrent stroke, thrombocytopenia, and neurological manifestations resembling those of MS are possible [44].

As the figures show, not all MS patients show elevated platelet activation or platelet-associated IgM. Since this study was not longitudinal, it is not known if the patients with elevated values represent a subset of MS patients, or if most MS patients have occasional or episodic elevations, possibly preceding exacerbations.

The classic neuropathology of MS consists of the perivenular plaque surrounding venular endothelial cells with infiltrating lymphocytes and macrophages [45]. The interactions of these cellular elements and resulting myelin and axonal damage are complex and incompletely understood. Our findings collectively suggest a complex interplay of platelets with endothelial and other cells in the pathogenesis of the MS plaque. The interaction of CD62p, PMP, macrophages, anti-phospholipid antibodies, and the CNS venular endothelium requires further study.

## Conclusion

Findings of this study demonstrate that platelets are significantly activated in MS patients. However, their role in pathogenesis of MS remains unknown. Further longitudinal studies of larger cohorts of MS patients are required to validate the significance of the present study and demonstrate the role of platelet activation in neuro-pathogenesis of MS.

## Abbreviations

MS: Multiple sclerosis; ITP: idiopathic thrombocytopenic purpura; PMP: plasma platelet microparticles; CD62p: P-selectin expression; CNS: central nervous system; PAg: platelet micro-aggregates; FITC: fluorescein isothiocyanate; CD62p: P-selectin; TF: tissue factor; PAF: platelet activating factor; PF3: platelet factor 3; CD31/PECAM-1: platelet endothelial cell adhesion molecule-1; PSGL-1: P-selectin glycoprotein ligand-1; PAF: platelet activating factor; PRP: platelet-rich plasma; PPP: platelet-poor plasma.

## Competing interests

The authors declare that they have no competing interests.

## Authors' contributions

WAS, WH, YSA and AM designed the clinical research protocol, provided expertise for laboratory measurement of platelet activity markers.

WJ and LLH contributed to the manuscript by collecting laboratory data, providing expertise for analysis of the obtained laboratory data, and doing biostatistical analysis and generating figures.

JSA contributed by preparing the manuscript and providing expertise in interpreting the obtained data on platelet adhesion molecules.
